# Sleep Deprivation in Middle Age May Increase Dementia Risk: A Review

**DOI:** 10.7759/cureus.37425

**Published:** 2023-04-11

**Authors:** Irina Balan, Nataliya Bilger, Dosbai Saparov, Ihor Hryb, Azamat Abdyraimov

**Affiliations:** 1 Geriatrics, Montefiore Medical Center, Wakefield Campus, Bronx, USA; 2 Clinical Simulation Center, Penn State University College of Medicine, Milton S. Hershey Medical Center, Hershey, USA; 3 Internal Medicine, Brookdale University Hospital Medical Center, Brooklyn, USA; 4 Neuroscience, University of Minnesota, Minneapolis, USA; 5 Biostatistics and Epidemiology, Ala-Too International University, Bishkek, KGZ

**Keywords:** all-cause dementia, middle age, short sleep duration, alzheimer’s dementia, sleep deprivation

## Abstract

Neurodegenerative diseases present increasing interest in clinical practice for the aging population and involve dysregulation of sleep-wake behaviors. Approximately 5.8 million adults aged 65 and older were living with Alzheimer’s disease (AD) in the United States in 2020 with increased mortality compared to the declining cardiovascular and cancer death rates. We conducted an extensive literature review to evaluate and synthesize evidence regarding the association between short sleep duration or sleep deprivation and the risk of developing all-cause dementia and Alzheimer’s disease. There are multiple mechanisms describing brain damage, such as brain hypoxia, oxidative stress, or blood-brain barrier (BBB) impairment, induced by chronic sleep restriction (CSR) and the potential correlation with future cognitive decline and dementia. More studies are necessary to identify the specific factors involved in the sleep loss-cognitive decline association that could be taken into consideration while elaborating recommendations for dementia prevention measures.

## Introduction and background

According to the International Work Group, Alzheimer’s disease (AD) is conceptualized as a clinical-biological syndrome, comprising typical amnestic features, age-related cognitive and functional decline, and neuropathological biomarkers [1,2]. In 2020, based on a Centers for Disease Control and Prevention (CDC) report, an estimated 5.8 million individuals aged 65 years or older in the United States had Alzheimer’s disease, and their death rates were higher than the decreasing rates of cardiovascular disease and cancer [3].

Neurodegenerative diseases present increasing interest in clinical practice for the aging population and involve dysregulation of sleep-wake behaviors caused by specific pathophysiological processes within the brainstem and hypothalamus [4]. Multiple observational studies with a follow-up of fewer than 10 years have revealed the possible association between mild cognitive decline and dementia and long or short sleep duration [5]. Most of the studies with follow-ups of 10 years and longer included respondents aged 65 years and older, impeding the analysis of the likelihood of developing dementia with early-life sleep deprivation or disturbances [5].

As reported by the CDC, short sleep duration (less than seven hours) was less prevalent among the respondents aged 65 and older (26.3%) compared with other age groups (18-64 years ranged from 32.2% to 35.6%), and there is no difference between males and females [6]. A higher age-adjusted prevalence was noted among American Indians/Alaska Natives (40.4%), multiracial non-Hispanics (44.3%), non-Hispanic blacks (45.8%), and Native Hawaiians/Pacific Islanders (46.3%) in comparison with non-Hispanic whites (33.4%), Hispanics (34.5%), and Asians (37.5%) [6]. Geographical distribution of short sleep duration varies among the United States with the highest proportion in the southeastern and the states along the Appalachian Mountains [6].

Consequently, we conducted a literature review to evaluate and synthesize evidence regarding the association between short sleep duration or sleep deprivation and the risk of developing all-cause dementia and AD.

## Review

Due to the lack of efficient treatment of dementia, the identification of modifiable risk factors has become imperative, considering the suggestive evidence of a correlation between dietary patterns, physical activity, sleep dysfunction, and cognitive impairment [7,8]. Multiple observational trials revealed the possible association between the decline of cognitive performance and sleep duration; however, the overadjustment of potential factors and confounding aspects limited the generalizability [8].

Sleep is essential for the revitalization of the functions of the body, including muscle restoration and hormonal regulation [9]. Sleep deprivation, sleep loss, and chronic stress have become a routine in our contemporary society and could induce increased performance variability and reduced response speed [10]. Zunzunegui et al. [11] revealed suggestive data of reduced axonal regeneration, synaptophysin expression, and ischemia-related vascular or neuronal cell growth in rats, showing the role of insufficient sleep in the regulation of the recovery processes and neuroplasticity after brain injury [11].

Mechanisms triggered by sleep disruption

The Impact of Disrupted Sleep on the Hippocampus

Multiple studies have shown the role of sleep in memory consolidation and enhanced hippocampus-dependent signaling [12]. The most susceptible to sleep deprivation is the hippocampus, which could be affected by modified protein synthesis, cellular signaling, and neuronal connectivity [13]. A group of researchers from Iowa, USA, have highlighted the impact of insufficient sleep on the remarkable downregulation of gene expression, such as cell adhesion molecules, involved in sleep control [13]. Subsequently, acute sleep deprivation influences transcription and translation in various cerebral regions, including the reduction of hippocampal plasticity [13]. Conclusively, sleep loss is positively correlated with hippocampal volume reduction and impaired attention, verbal and nonverbal memory, and frontal lobe function [14].

The Impact of Disrupted Sleep on the Glymphatic System

It was demonstrated that sleep deprivation could be responsible for increasing levels of soluble amyloid Aβ in animal and human models and for the augmentation of amyloid plaque deposition in mice models [15]. Many investigators showed the significance of the glymphatic system, a perivascular network for cerebrospinal fluid (CSF), in removing toxins from the central nervous system (CNS) [15]. Glymphatic clearance is a continuous process occurring during wakefulness and sleep [16]. An increase in clearance was particularly noted during the non-rapid eye movement stage of sleep (N), associated with an influx of CSF in the interstitial cavities during the slow-wave sleep (N3) [16]. Therefore, a functional glymphatic system is crucial for the homeostasis of CNS and the prevention of neurodegenerative diseases [17].

The Impact of Brain Hypoxia

Severe hypoxia involves brain tissue damage by decreasing oxidative phosphorylation and other oxygen-dependent processes caused by chronic disorders such as obstructive sleep apnea or environmental conditions [18]. Sleep deprivation and cerebral hypoxia could contribute to cognitive impairment in patients with sleep apnea [19]. Gozal et al. [20] demonstrated the association of intermittent hypoxia with impaired spatial learning in adult rats and boosted apoptosis in the cortex and first region of the hippocampus (CA1). Regardless of the well-known negative effects of intermittent hypoxia, there is increasing evidence revealing possible neuroprotective characteristics of moderate and episodic brain hypoxia [21].

The Impact of Oxidative Stress

The cognitive decline in Alzheimer’s disease is caused by an impaired neuroendocrine system, glucose metabolism, and oxidative stress [22]. Oxidative stress is a state described by the inequality between oxidative and antioxidant combinations due to the disproportionate production of free radicals or a lack of the ability to decrease them, leading to the oxidation of biomolecules, failing their biological processes and generation of homeostatic damage that can affect the cells, tissues, and organs [23]. Antioxidants belong to structurally heterogeneous groups that share the ability to scavenge free radicals and form the first line of protection against possible harm induced by reactive oxygen species (ROS) [24]. ROS is an extensive term that contains free radicals (superoxide, hydroxyl, and nitric oxide) and other non-radical species derived from oxygen (hydrogen peroxide), causing lipid peroxidation (LPO), protein oxidation, and deoxyribonucleic acid (DNA) injury and, subsequently, resulting in cell death [25,26].

Sleep is a dynamic resting condition with antioxidant effects, accountable for eradicating the ROS generated during wakefulness [27]. Studies showed a decrease in total antioxidant capacity and augmentation of oxidative stress markers after continuous hours of shift work [28]. Sleep deprivation involves an oxidative challenge for the brain; therefore, it could be defensive against oxidative injury [29]. The research of Swarnkar et al. [30] assessed oxidative stress in the whole-brain homogenates of sleep-deprived rats. It demonstrated differences in specific brain regions linked to oxidative stress, such as reduced glutathione levels in the hippocampus and thalamus.

Night shift workers with an evident lack of sleep might develop elevated oxidative stress damage induced by an increase of ROS production and a reduction in enzymatic (catalase (CAT) and superoxide dismutase (SOD)) and nonenzymatic ferric reducing antioxidant power (FRAP) antioxidant protection [31]. Furthermore, the biological rhythm disorder resulting from the night shift shows disturbance of the hypothalamic-adrenal-pituitary axis and its mediator, cortisol, alterations in glutathione levels, and methylation of DNA [32]. Nevertheless, Ulas et al. [33] discovered that the increase in oxidative stress parameters observed after the night shift in medical workers was not impacted by the assignment or role at work but rather by a lengthy work activity and insufficient rest duration.

The study of Teixeira et al. [34] determined the oxidative stress damage induced by sleep deprivation in night workers caused by the imbalance of pro- and antioxidant factors. Melatonin exhibits antioxidant potency and is a possible eliminator of ROS [35]. A low level of circulating melatonin during night work, not adequately compensated by the sleep duration during the day, explains the association of oxidative stress with the circadian rhythm disturbance caused by night work [35]. The mitochondrial DNA has elevated susceptibility to oxidative stress, regardless of its extensive antioxidant system [36]. The aging brain, specifically in Alzheimer’s and vascular dementias, is particularly described by mitochondrial DNA and progressive oxidative damage [36].

Circadian Activity Rhythm Disturbances

Several animal models showed that the modifications of sleep-wake cycles could be linked to the increased levels of hyperphosphorylated tau proteins in the brain and, subsequently, of extracellular amyloid, the main component of Alzheimer’s disease [37]. There is increasing evidence showing that circadian rhythm abnormalities start early in Alzheimer’s disease and may play a role in the initiation of the disease [38]. Musiek [38] revealed that sleep and circadian rhythm disturbances are common in AD patients, notably as the condition progresses. A cohort study revealed that the circadian rest-activity pattern may affect the prediction of cognitive impairment in mild and moderate AD [39].

Disrupted circadian rhythm measures, including delayed timing of peak activity on wrist actigraphy, less vigorous rhythm, and lower amplitude, were determined to be predictive of the future development of mild to severe cognitive impairment in cognitively normal females [40]. Circadian dysfunction impairs cholinergic signaling, which could be considered a factor linked to cognitive decline and dementia [41]. Ruby [41] developed animal models and demonstrated the impact of disrupted circadian rhythms on the impairment of object recognition, social memory, and the immune system.

Therefore, understanding the influence of sleep and circadian disturbances in healthy middle-aged people on the pathogenesis of dementia and AD may help the identification of modifiable risk factors to delay their onset and the development of new therapies [42].

Overexpression of Orexins

Small groups of neurons in the lateral hypothalamic and perifornical areas produce a neuropeptide, orexin, also known as hypocretins, that are responsible for the regulation of sleep, wakefulness, feeding, and energy homeostasis [43-46]. Overexpression of orexins leads to non-rapid eye movement (non-REM) sleep fragmentation with an occasional reduction in REM sleep [47].

Hypothetically, the typical AD sleep disturbances, including sleep fragmentation, nocturnal arousals, diurnal somnolence, and REM sleep impairment, are associated with orexinergic neurotransmission dysfunction [48]. Liguori et al. [49] assessed the CSF orexin in AD and revealed a positive correlation between t-tau protein and CSF orexin levels in moderate and severe AD, but no evident correlation in patients with mild AD. Subsequently, some clinical trials showed potential beneficial effects on sleep in AD patients of the orexin antagonists; however, further studies are necessary to evaluate the long-term benefits of this pharmacotherapy [50].

Blood-Brain Barrier Impairment

The blood-brain barrier (BBB) consists of specialized microvessels that filter harmful substances from entering brain tissue that could affect sleep and cause sleep disorders [51,52]. Unfortunately, there is insufficient information regarding the regulation of the BBB and its importance in CNS homeostasis [53,54]. The BBB also regulates regional blood flow and metabolism, according to a new study on gliovascular coupling [55]. 

Sleep deprivation causes the disruption of circadian rhythms, as well as the production of new molecules, such as free radicals and cellular metabolites [56]. Arousal from sleep could induce an increase in pulse pressure and vascular shear stress, resulting in cerebral perfusion modifications [57].

Pan and Kastin [58] designed two animal models that showed the BBB alterations produced by sleep disorders, including chronic sleep restriction (CSR) and sleep fragmentation procedures, in order to imitate the human situation of chronic sleep loss and disturbance. In the BBB microvessels, multiple tight junction proteins showed decreased production at both the mRNA and protein levels, which was consistent with the slight increase in paracellular permeability [59]. Glucose uptake was also reduced in all assessed brain and spinal cord regions, being correlated with the reduction of Glut1 transporter expression in the BBB microvessels [59].

Several studies have found that sleep deprivation is responsible for systemic low-grade inflammation, which is characterized by the release of various molecules, including chemokines, cytokines, and acute-phase proteins, affecting the brain endothelial cells [60].

Vascular reactivity may have been reduced by chronic sleep restriction (CSR) because the BBB microvessels expressed lower levels of endothelin-1 and the inducible and endothelial forms of nitric oxide synthase [61]. These aspects contrast with higher levels of cyclooxygenase-2 and the chemokines C-C motif ligand (CCL)-7 and CCL-12, both of which are pro-inflammatory markers [57].

CSR increases COX-2-related inflammation, influences vascular reactivity, lowers glucose transporters, and impairs BBB permeability, although the BBB disruption generated by six days of CSR is restored after 24 hours of recovery sleep [59]. Specific cytoprotective mechanisms underlying these alterations might possibly be exploited to prevent synaptic malfunction and neurodegeneration in patients with sleep problems that continue for a long period [59].

Long-term sleep deprivation can cause low-grade systemic inflammation and impaired blood-brain barrier function, including a reduction in the number of solute transporters. This may have a detrimental effect on cognitive functioning and increase the risk of neurological and neurodegenerative illnesses. Consideration should be given to the role of inflammation in the breakdown of the blood-brain barrier brought on by insufficient sleep. When examining the effects of sleep deprivation, the systemic and local effects of chronic sleep loss should be taken into account.

## Conclusions

The main mechanisms involved in the brain damage induced by sleep deprivation are brain hypoxia, impairment of the glymphatic system and brain-blood barrier, circadian rhythm disturbances, orexin overexpression, and the impact on the hippocampus. A multitude of aspects should be considered while analyzing the potential correlation between chronic sleep restriction in middle-aged individuals with future cognitive decline and dementia. Further studies are necessary to identify the specific factors involved in the sleep loss-cognitive decline association that could be taken into consideration while elaborating the recommendations for dementia prevention measures and subsequent public health policies.
